# What Does Twitter Say About Self-Regulated Learning? Mapping Tweets From 2011 to 2021

**DOI:** 10.3389/fpsyg.2022.820813

**Published:** 2022-02-24

**Authors:** Mohammad Khalil, Gleb Belokrys

**Affiliations:** Centre for the Science of Learning & Technology (SLATE), Faculty of Psychology, University of Bergen, Bergen, Norway

**Keywords:** self-regulated learning (SRL), Twitter analysis, topic modeling (LDA), geocoding analysis, descriptive analysis, self regulation

## Abstract

Social network services such as Twitter are important venues that can be used as rich data sources to mine public opinions about various topics. In this study, we used Twitter to collect data on one of the most growing theories in education, namely Self-Regulated Learning (SRL) and carry out further analysis to investigate What Twitter says about SRL? This work uses three main analysis methods, descriptive, topic modeling, and geocoding analysis. The searched and collected dataset consists of a large volume of relevant SRL tweets equal to 54,070 tweets between 2011 and 2021. The descriptive analysis uncovers a growing discussion on SRL on Twitter from 2011 till 2018 and then markedly decreased till the collection day. For topic modeling, the text mining technique of Latent Dirichlet allocation (LDA) was applied and revealed insights on computationally processed topics. Finally, the geocoding analysis uncovers a diverse community from all over the world, yet a higher density representation of users from the Global North was identified. Further implications are discussed in the paper.

## Introduction

Self-Regulated Learning (SRL) has gained much attention recently. Researchers have presented theories of SRL in various contexts of modern educational models, including SRL in formal learning, SRL in informal learning, and SRL in non-formal learning settings. Most of these learning models have been shaped by the digital revolution of education (i.e., teaching and learning) through the introduction and usage of learning management systems, smart devices, Massive Open Online Courses (MOOCs), and other data-driven applications such as learning analytics. In addition, social media has emerged as a popular forum for learning and sharing information as well as discussing activities that are related to education, concepts, and classrooms (Clarke and Nelson, 2012). The term microblogging in social media forums is seen as a new form of blogging activity for the general public that enables rapid dissemination of information and exchange of artifacts and opinions among diverse communities.

Twitter is one of the most popular microblogging services that entails a vast corpus of contextual data. According to Ahlgren (2020), there are over 500 million tweets each day generated by 350 million active users. Twitter structure is simple. Users are allowed to tweet short messages that are only 280 characters in length (previously in the early times of Twitter, users were allowed to tweet only 140 characters). Twitter permits users to interact with microblogs in various ways: posting on one’s profile page (tweet), sharing a microblog on their profile (retweet), replying to someone’s microblog (reply), clicking the action button of a “heart” (like), a mention of someone (user hashtag), and linking to a context (topic hashtag).

In the scientific domain, Twitter has been actively used to raise scholarly discussions and exchange of scientific information (Darling et al., 2013). That is, research shows that scholars tend to use Twitter for sharing activities and providing quick and direct reflections on conferences, publications, and getting into debate (Collins et al., 2016). Relying on the increasingly respected practice of science communication with the public, evidence has been found on microblogging in general and Twitter in particular as a means for outreach and increasing science literacy (Parsons et al., 2014; Collins et al., 2016). Since Twitter is gradually becoming a venue for academic microblogging (Dhir et al., 2013; Collins et al., 2016), knowledge about research topics, interest and scholarly interactions are becoming immense, and “fortunately” automatically recorded. The availability of such a rich repository of data on research topics offers valuable insights to discover and understand trends within scientific domains (Chen et al., 2015). As an important theory in the field of education, SRL can benefit greatly from such an intuitiveness approach. In fact, understanding the discussions around SRL in the scope of one of the most popular social networking services can help identify themes and changes. In addition, the analysis can provide a determination of critical gaps and yet plan for future steps by involving a different voice from outside academia.

Recently, several studies have reviewed scholarly works on SRL (Winne, 2021; Yusufu and Shakir, 2021). Nevertheless, we do not know how the theory of SRL is discussed on social media in general and Twitter in particular. Whereas Twitter has been found to stimulate interest in certain topics (Han et al., 2021), no study reviewed Twitter to explore the SRL theory. We want to take advantage of this social media platform and offer an alternative approach to investigating and exploring SRL communication and discussion. Our exploration includes investigating Twitter conversations on SRL, the main topics of interest raised by the public discussion, and where do they originate from?

A big challenge of analyzing social media data is how to excerpt valuable insights from a large amount of data. However, the fast development of data science technologies allows to analyze a large amount of unstructured content data and gain insights in a short time (Blazquez and Domenech, 2018). In particular, and besides descriptive and content analyses, this work uses Natural Language Processing (NLP) techniques, including unsupervised methods and analyze 54,070 tweets collected in a time frame between 2011 and 2021.

Therefore, the main contribution of the paper is to potentially reflect on the SRL theory and reveal new insights about the community discussions on SRL by analyzing the Twitter microblogging data with particular key search terms. The exploration of Twitter data is not new, however, to our knowledge, this is the first study of such an approach on SRL to be conducted, demonstrating a gap of knowledge that should be tackled. As a result, this work brings in interesting findings to fill the research gap. First, the paper bridges the research gap on SRL by leveraging user-generated data which is commonly unfiltered information and uses a unique source other than those available from general derivations (i.e., scholarly publications, practices). Second, we extract some interesting topics on SRL from unsupervised topic modeling, including five main themes that could demonstrate a different direction on social media than what is used to account in academic shares. Last but not least, the article reinforces originality by bringing in geocoding, a technique that indexes particular information to a geographical position, and unveils that discussion on SRL is more prevalent in some particular geographical regions than others.

The remainder of the paper is structured as follows. In section “Related Work,” we discuss related work. Section “Theoretical Framework” covers the theoretical framework that shaped our understanding of the research problem and analyses. Section “Methodology” and “Results” draw insights into the used methodology and results, respectively. Section “Discussion” discusses the results and bring in our answer to the research questions. Finally, the paper concludes with implications, limitations, and future directions.

## Related Work

Self-regulated learning is a skill of self-thought, plan, and action that has been identified as one of the critical factors affecting student success in learning processes (Zimmerman, 1990; Winne, 2021; Yusufu and Shakir, 2021). While there are various models of SRL, most of the models agreed that SRL is cyclical and clustered into three phases, namely forethought, performance, and reflection. One of the grounds for the relevant interest in SRL is the growth of digital, online, and virtual courses in the context of formal and informal learning environments (Lim et al., 2021). The reason of which returns to the students who are in needed skills to “actively make decisions on the metacognitive and cognitive strategies they deploy to monitor and control their learning to achieve their goals” (Lim et al., 2021, p. 2). SRL strategies such as goal setting, time management, and help-seeking are useful and common practices used to explore and investigate SRL processes (Yusufu and Shakir, 2021).

Encouraging online collaborative activities through social media platforms to seek help from other colleagues was identified as relevant and essential for SRL (Yen et al., 2021). Yen et al. (2021) also found that blogging on social media effectively engages students in self-evaluation and self-reflection, which, as mentioned earlier, are fundamental parts of the SRL phases. With that in mind, social media may encompass important discussions on the theoretical and practical approaches for better self-regulation.

Recently, there have been a growing number of Twitter-related research works. Some of the studies powered up Twitter and used the huge collection of microblogs contextual data to address interesting research questions. For example, Chen et al. (2015) analyzed tweets of 4 years period of the official learning analytics and knowledge conference to gain insights into the community. The analysis revealed that Twitter was helpful to identify trends of learning analytics as well as identify major personal experiences. Chen et al. (2015) were able to characterize an escalating trend of student-centered topics on engagement and assessment as well as cluster tweets into topics using topic modeling to show the diversity of the field of learning analytics.

The conversational nature of Twitter has been identified to be useful in detecting user networks to discover scientific knowledge across different communities. The study by Díaz-Faes et al. (2019) provided novice evidence on Twitter studies to break new ground for systematic analysis around science. Díaz-Faes et al. (2019) analyzed over 1.3 million unique users’ data and 14 million tweets on scientific publications to outline the general activities of Twitter communities and their interactions with scientific outputs based on social media metrics. Some of the major findings of their study has revealed the significant disciplinary differences of how researchers behave in the social media realm and the development of scholarly identity of researchers.

Another example is the study by Garcia and Berton (2021) who used sentiment analysis to explore a large number of tweets in Brazil and United States on related microblogs to COVID-19. The researchers identified a general negative emotions dominancy during the COVID-19 pandemic for almost all the topics in United States and Brazil. A key contribution of Garcia and Berton (2021) study was enriching the library of the Portuguese language with keywords related to positive and negative emotions as well as gap the literature with new sentiment content for the development of new techniques for processing languages other than English.

Perhaps some of the most popular analysis methods of Twitter from the literature are content analysis and topic modeling (Giachanou and Crestani, 2016). The latter method has been immensely used to identify topics from complex yet short textual data. One interesting example of how topic modeling has been used with a large tweets database is the study by Dahal et al. (2019). The researchers were able to infer different topics of discussion on the issue of climate change and how it is perceived by the general public. Dahal et al. (2019) found that the discussions of climate change in the United States are less focused on policy topics than other countries in Europe. Other examples from the literature used topic modeling to examine themes discussed on Twitter about the COVID-19 pandemic (e.g., Boon-Itt and Skunkan, 2020; Wicke and Bolognesi, 2020).

Topic modeling helped Saura et al. (2021) divide a large corpus of nearly 900,000 tweets on security issues in smart living environments. The result of this study identified 10 topics related to privacy and security breaches and smart living environments such as the Internet of Things. One of the significant implications that took advantage of Twitter microblogs using topic modeling is identifying key concerns raised by users. For example, Saura et al. (2021) determined that malware, data cloud storage, and cyber-attacks are among the major issues Twitter users reported and require further attention by manufacturers.

With respect to content analysis, Twitter offers various possibilities, for example hashtag analysis. Hashtags enable users to identify other users based on their interest in parallel topics (Kimmons et al., 2017). As such, hashtags provide sharing of information in an organized manner with which resources are curated based on shared interest. Another research study by Kimmons et al. (2021) examined tweets that incorporate a hashtag of #EdTech, found out that discussions of educational technology have been changing with the present pandemic. It seems that the COVID-19 has triggered the emerging usage of new terms in educational technology such as “remote learning.” The study by Kimmons et al. (2021) also stated that trends of educational technology (i.e., EdTech) had been largely influenced by a small group of active Twitter users during the time of the pandemic.

A less popular but interesting analysis method is location analysis based on microblogs (i.e., geocoding). Using social media data for geographical research can be used to identify trends, explain patterns and describe various geographical phenomena (Goldberg, 2008). In Twitter, researchers have used geolocation analysis to map the felt area by earthquakes by examining the tweets generated after a particular time (Earle et al., 2010). Others used geolocation to identify accidents reported on Twitter in large cities (Milusheva et al., 2021).

In general, we learned from the literature that exploring the public discussions surrounding the SRL theory using Twitter analysis methods could offer useful information and present alternative perspectives to the theory. Provided that, the current study aims to gain a broader understanding of how the SRL theory is discussed in the public affinity space and how it has been argued over the last 10 years. To achieve this goal, our analyses will attempt to answer the following research questions:

• What are the general characteristics of Twitter conversation on SRL?
• What are the main topics of interest that are related to SRL from Twitter public discussion?
• Where do English-based SRL discussions originate from?

## Theoretical Framework

Our understanding of investigating SRL using Twitter is grounded in Gee’s (2012) theoretical framework of *affinity spaces*. Gee identifies affinity spaces when typical geographical boundaries are humbled. He conceives spaces as physical, blended or digital spaces where individuals share common interests and endeavors. In these spaces, individuals also communicate and interact with each other. Unlike traditional contexts, affinity spaces provide wide areas for involving individuals that are open for everyone.

According to Carpenter et al. (2020) study, the phenomena of infinity space can be articulated on social media, and Twitter is one of these. In the context of this paper, we follow a similar approach and use “Twitter space” and “Twittersphere” interchangeably to link to Gee’s grounding of *affinity spaces*.

## Methodology

As stated earlier, the objective of this research study is to carry out analyses on Twitter tweets with a particular emphasis on the SRL theory from the time span of 2011 till 2021. As a consequence of this study goal and after the data collection, we performed key steps to clean and prepare the dataset. We then followed three main methods to answer the research questions, descriptive analysis, geocoding analysis, and topic modeling.

In the context of this work, the term “tweet” refers to a microblog message from a Twitter account that consists of a limited number of characters, 140–280 characters. The term “organic tweet” refers to original microblog tweets, while a “retweet” means a re-post of a tweet that is shared among one’s followers. In this paper also appears terms of “hashtags” and “likes.” The hashtags are words that start with “#” and when used by an author, it becomes linked to other tweets that share-alike. Finally, “likes” are Twitter interactions that are represented by a small heart referring to one’s appreciation for a particular tweet.

### Data Collection

The data collection process was carried out using the well-known programming software, Python with scripts that belong to the standard indexed libraries^1^ (e.g., json, requests, os, and time). To retrieve the tweets from Twitter, we used the Twitter Application Programming Interface of (API) using private tokens and keys. The majority of the API functions were optimized to pull out the needed raw data from Twitter database (e.g., text, likes, retweets, hashtags, etc.) to proceed with the analysis.

In order to retrieve the needed information, we created a corpus of search terms directly connected to SRL as the following:

keywords = “self_regulated_learning” OR “selfregulatedlearning” OR “self-regulated learning”

The return results include those tweets that use the trigram word of “self-regulated learning” with and without hyphen, underscore, dash…etc., so-called regex check.^2^

In addition, we search a numerous number of hashtags that could be linked to SRL as the following:

This large corpus of hashtags was constituted based on screening particular academic article keywords relevant to SRL. Later in the study, we will investigate whether some of these hashtags are among the top discussed by the public on the Twittersphere.

Provided that the Twitter API has a quota of 900 tweets per 15 min, the automated process of retrieving the whole dataset of the tweets took around 37 h. The time frame of the search for the tweets is 10 years. That is, the exact date is between January 1, 2011, to September 30, 2021. The total number of retrieved tweets from the search terms within the specified time period is 54,070 tweets. These tweets are posted by 9,951 unique authors and interacted (i.e., likes, retweeted, etc.) by a population size of 29,556 users.

keywords = “(#selfregulatedlearning OR #learning OR #education OR #metacognition OR #elearning OR #edchat OR #highered OR #learninganalytics OR #edtech OR #teaching OR #srlcanada OR #university OR #universidad OR #teachertraining OR #onlinecourse OR #motivation OR #selfregulation OR #mooc OR #moocs OR #teachers OR #onlinelearning OR #srl2 OR #students OR #assessment OR #activatedlearning OR #blendedlearning OR #feedback OR #pedagogy OR #formativeassessment OR #metacognitive OR #technologies OR #teacher OR #middleschool OR #teach OR #hybridlearning OR #selfreg OR #reflection OR #agency OR #flippedlearning OR #analytics OR #technology OR #self_regulated_learning OR #bigdata OR #teachingandlearning OR #educators OR #selfregulatinglearning OR #training OR #student OR #edpsych OR #computers OR #behavior OR #learner OR #mlearning OR #multimodal OR #experientiallearning OR #collaboration OR #data OR #psychology OR #personalizedlearning OR #selfreflection OR #asynchronouslearning OR #flipclass OR #highereducation OR #dashboards OR #independentlearning OR #remotelearning OR #digitallearning OR #learninganddevelopment OR #flippedclassroom OR #lifelonglearning OR #academic) AND (“self-regulation” OR “self-regulated” OR “self-regulate”)”

### Data Cleaning and Preparation

Similar to many studies, such as Dahal et al. (2019), we carried a substantial work to process and filter the tweets before applying the Twitter analysis. To extract and clean the tweets from the stop words, which is a common practice in microblog analysis, we used the Natural Language ToolKit (NLTK) library. Such removed stop words from the corpus are “and, or, has, have, are, is, etc.” In addition, we used the Python regex to remove emojis and digital expressions like dash and underscore.

It is also common in microblogs to include short words. For that reason, we exclude those that are less than four letters. However, and to preserve popular short academic abbreviations related to SRL and education, we created a whitelist that includes several short words (see section “Topic Modeling” for examples). This whitelist is created manually by scanning the top 200 short words from the retrieved tweet dataset. Empty sentences and duplicate records have also been removed. To align well with our universal Twitter analysis in this study, we removed so-called low TF-IDF (term frequency-inverse document frequency) as recommended by Tajbakhsh and Bagherzadeh (2016). Further data customizations were done to fulfill our needs for the geocoding and the topic modeling analyses. More details are provided in the sections below.

### Descriptive Analysis

The first analysis method used in this study is descriptive analysis. This analysis includes further investigation of listing top tweets, number of tweets, source of tweets, language used, common tweet words (i.e., word cloud), number of likes, retweets and hashtag analysis.

### Geocoding

Geocoding is the procedure of indexing a description of particular information that can be linked to a geographical position on a world map (Fatima et al., 2021). There are several advantages of using geocode analysis such as identifying trends and explaining patterns based on geographical phenomena. While Twitter made it possible for users to enable their location when tweeting, not so many tweeters used that function. For that reason, in June 2019, Twitter decided to stop users from tagging their locations.^3^

The form when users enable the location of their tweets is called geotagged tweets. In this paper, we could not identify more than 15% of locations based on geotagged tweets. To overcome that, we followed the direction of what is so-called geotagged users referring to our ability to extract the location of users based on their self-reported position.

To display a world map of users who used the terms of SRL, we needed to distill the names of cities and countries from the self-reported profiles (*N* = 29,556) and then identify their geographic location. Such a process can be complicated because Twitter offers users to freely designate their place of living instead of choosing a country/city from a pre-selected list. For example, some users could state that their place of living is Germany; some other users may state that Berlin is their place of living. For humans, this is easily understood but not for machines. To surpass this issue, we had to follow Natural Language Processing (NLP) techniques.

We used several computational solutions to carry out the NLP techniques for the geocoding analysis. For purposes of tokenization of texts, we used spaCy.^4^ For purposes of obtaining geographical coordination of countries, we deployed a local geocoding service called Nominatim^5^ to identify locations on the world map. Furthermore, we employed Nominatim docker^6^ to speed up the process of identifying positions on Earth.

### Topic Modeling

The third method of analysis in this study is topic modeling which is a common text mining technique used to discover hidden semantic of textual corpora (Blei et al., 2003). There are several algorithms in topic modeling, nevertheless, the unsupervised modeling of Latent Dirichlet Allocation (LDA) considered to be one of the most popular ones that has been widely used in social sciences (ibid). In the light of its simplicity and efficiency, we used LDA to extract relevant themes and topics through document collections of the tweets. To do the LDA, we first had to identify the number of topics, which could be done by several approaches. In this work, we picked the coherence method to calculate the consistency of topics and validate the optimal number of generated topics (Stevens et al., 2012).

Before doing the topic modeling analysis, we cleaned and filtered the tweets as described before in the Data Clearning and Preparation section. Moreover, the following steps were performed:

• Applied two dictionaries to improve topic extraction, blacklist of words (e.g., today, yesterday, look, will) and white list (e.g., SRL, MOOC, AI) of words.
• Removed non-English tweets.
• Performed light lemmatization, which is a technique to return the verbs and words to the base form. The reason is to analyze different forms of words by a single item instead of several.
• Cleared tweets from hashtags so that the topic modeling is not affected by retweets.
• Excluded retweets to reduce generating biased topics.

After the cleaning and filtration process, we imported the refined dataset into Python and ran the topic modeling algorithm using Gensim’s LDA (Rehurek and Sojka, 2011) and built the model.

### Privacy Consideration

The data collection in this study used the Twitter API, which prevents mining any private and protected information. We stress two main points that define our commitment to privacy and data protection consideration:

• Twitter is a public social networking service where users cast their microblogs online and therefore the data collected by the Twitter API is considered “public data” (Deacon et al., 2021).
• The collected Twitter data has not been engineered to extract other than the published information by the users.

## Results

### Descriptive Analysis

**FIGURE 1:**
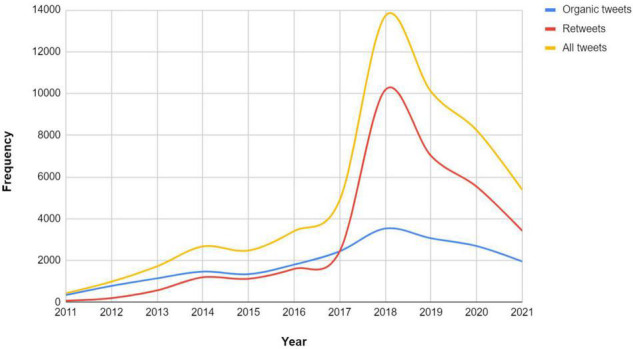
Breakdown of all the retrieved tweets (yellow line), original tweets (blue line) and retweets (red line) of SRL (2011–2021). Best viewed in color.

The first analysis method conducted is descriptive. The descriptive analysis provides a general overview of the dataset in terms of the tweet count, tweet sources, number of likes and retweets, hashtags used, and the language of each. With respect to the number of tweets, Figure 1 shows a breakdown of the counts per year. The *x*-axis shows the time span between 2011 and 2021, and the *y*-axis shows frequency. It is observed that there is a steady increase in the number of tweets for the time period between 2011 and 2017. A strong spike in the mined tweets happened between 2017 and 2018. However, we see a dramatic decrease after 2018.

To get an insight into the number of tweets and retweets, we depict a line graph of the two, as also shown in Figure 1. In total, there are 20,647 organic tweets and 33,423 retweets in the dataset. Aligning with the number of the general tweet stats, both the number of organic tweets and retweets has been steadily increasing in the first 6 years. In a parallel manner, organic tweets keep a continuous growth in 2018 while a strong spike in the number of retweets is clearly visible in that particular year. In the time period of 2019–2021, we record a decreased traffic of SRL tweets and retweets.

**FIGURE 2:**
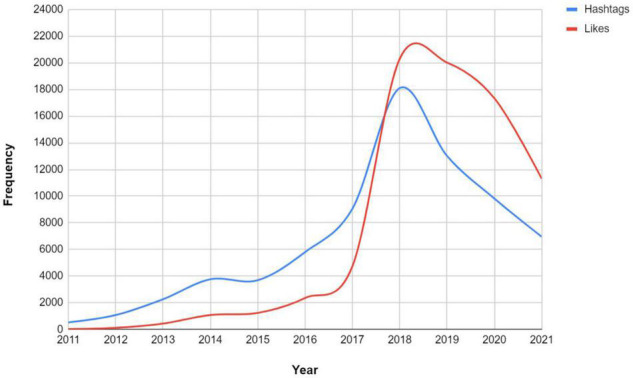
Comparison of likes and hashtags of tweets on SRL (2011–2021).

Next, Figure 2 depicts the distribution of likes and hashtags of the collected tweet dataset on SRL. In total, there are 74,106 hashtags and 72,631 likes used. Overall, there has been a growing number of both till 2018. The number of likes exceeded the hashtags in 2018. Nevertheless, the number of likes and hashtags started to decrease after 2019 till 2021.

Table 1 shows a breakdown of the average number of retweets, likes and hashtags per year.

**TABLE 1:** Breakdown of the average number of retweets, likes, and hashtags.

Year	Average number of retweets	Average number of likes	Average number of hashtags
2011	0.2436	0.0774	1.2118
2012	0.2608	0.1265	1.0894
2013	0.4996	0.258	1.3135
2014	0.8158	0.406	1.4057
2015	0.8358	0.5008	1.4895
2016	0.8872	0.6924	1.6998
2017	0.9988	0.9685	1.8507
2018	2.8765	1.4793	1.3196
2019	2.2856	1.9839	1.2899
2020	2.0541	2.1038	1.1916
2021	1.7517	2.1003	1.2917

Following that, we looked at the data sources of our Twitter dataset. Out of the 54,070 tweets, we were able to identify 87% of the tweet sources which are broken down in Table 2. Around 50% of the Twitter sources belong to mobile phone systems (i.e., iPhone and Android). The usage to tweet on SRL directly from a web client equals 12.4% of the total quota. Interestingly, around 12% of the tweets used to blog on Twitter came from tweet management applications such as TweetDeck, Buffer, and Hootsuite, which are commonly used to schedule tweets and connect with other social networking services than Twitter. The rest of the identified tweet sources come from the Twitter app on the iPad system.

**TABLE 2:** Top 10 sources of the tweets.

Source	Number of tweets
Twitter for iPhone	18,588
Twitter for Android	7,695
Twitter Web Client	6,719
Twitter Web App	4,835
Twitter for iPad	3,006
TweetDeck	1,867
Hootsuite	1,331
Buffer	1,286
Hootsuite Inc.	1,011
SocialOomph	912

Furthermore, we looked at the hashtags that were populated by the community. Table 3 shows the top 5 hashtags per year. The use of #edchat is observed to be very popular in the early years. When investigated, #edchat is a hashtag that encompasses a small part of education community blog that is interested in making learning better for kids.^7^ In the last couple of years, hashtags of #selfreg and #selfregulation were dominant in the context. Other interesting used hashtags in the community are conference hashtags (e.g., #icalt2011, #change11), disciplines (e.g., #education, #psychology), primary and secondary education (e.g., #children, #70playactivities, # sd61learn), and educational technology (EdTech) communities (e.g., #edtech).

**TABLE 3:** Top 5 hashtags per year.

Year	Hashtag (frequency of occurrences)
2011	#edchat (28), #psychology (24), #children (22), #icalt2011 (18), #elearning (16)
2012	#edchat (116), #edtech (56), #education (37), #elearning (36), #change11 (21)
2013	#edchat (182), #bigdata (162), #education (116), #teachers (55), #srlcanada (52)
2014	#edchat (566), #education (114), #bced (107), #sd61learn (92), #marketing (87)
2015	#edchat (286), #psychology (109), #bced (108), #sd16learn (95), #education (73)
2016	#education (406), #70playactivities (358), #edchat (194), #children (134), #therapy (98)
2017	#education (1070), #70playactivities (912), #children (335), #schoolpsych (269), #edchat (233)
2018	#education (1092), #edchat (927), #edtech (720), #elearning (644), #ukedchat (466)
2019	#edchat (360), #education (290), #exercise (248), #metacognition (244), #learning (232)
2020	#education (307), #metacognition (225), #teaching (215), #edchat (180), #cpd (159)
2021	#education (174), #learning (128), #edchat (120), #metacognition (101), #alratv (73)

Given that the raw dataset we have is multilingual, another aspect we used of the descriptive analysis is a synthesis of the tweets’ language on SRL. As expected, the most used language is English (97.6%), followed by Indonesian (0.56%), Spanish (0.37%), and Dutch (0.23%). The Twitter API could not identify some tweets languages (0.56%). The rest of the used languages (0.82%) belong to Japanese, Russian, French, Swedish, Catalan, Arabic, Filipino, German, Finnish, Portuguese, Danish, Romanian, Korean, Hindi, Norwegian, Turkish, Polish, Bulgarian, Latvian, Estonian, and Hebrew. These count for 390 tweets only.

**FIGURE 3:**
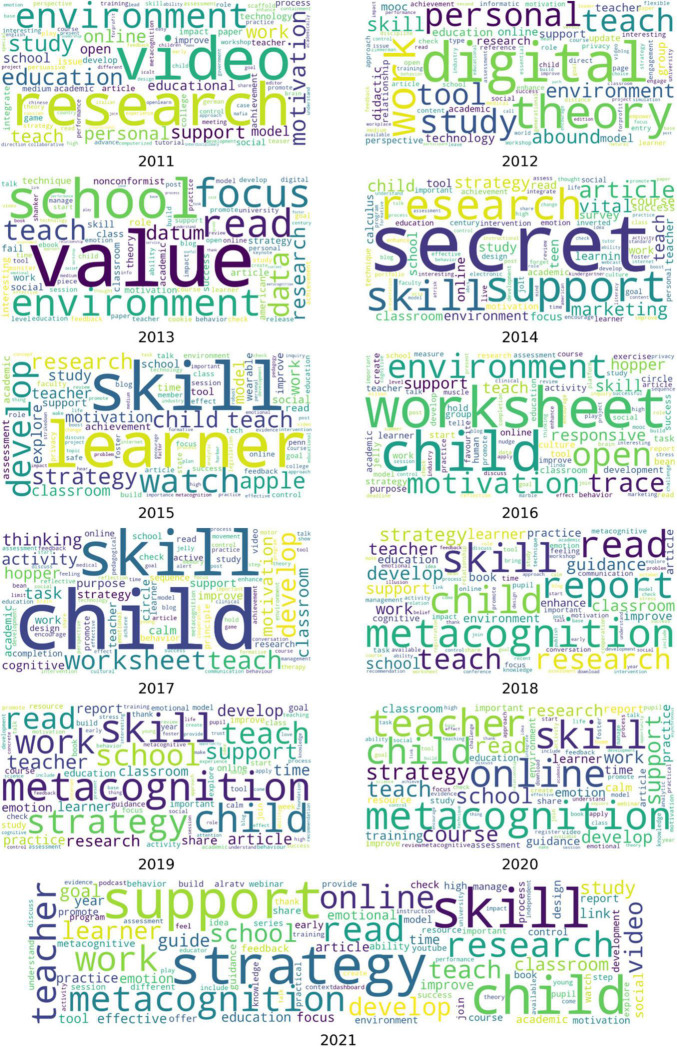
Word cloud of the most common words in the corpus of 54,070 tweets on SRL discussions divided by year (the larger the word print, the more frequent it occurs).

At the final stage of the descriptive analysis, we looked at the content. Table 4 presents some examples of public tweets used from the SRL tweets pool. While content varies like any other discussion board, we decided to create a word cloud of each year’s tweet bundle without any computational algorithmic correction except processing the corpus for data filtration as presented in section 3.2 (see Figure 3). The greater the size of the word appears in the figure, the greater occurrences exist in the corpus. In 2011, words “research, video, and environment” appear more often than the other. In 2012, “digital and theory,” in 2013, “value, school, and focus,” in 2014, “secret, support, and research,” in 2015, “skill, learner, and watch,” in 2016, “worksheet, child, and environment,” in 2017, “child and skill,” in both 2018 and 2019, “metacognition and skill,” in 2020, “metacognition, online, and teacher,” and finally in 2021, “skill, strategy, and support.”

**TABLE 4:** Selected high rating (i.e., in terms of like, retweet, and quote counts) tweets from the dataset.

Year	Tweet
2011	By dgasevic: “Best paper award for our paper “A Semantic Web-enabled Tool for Self-Regulated Learning in the Workplace” at #icalt2011”
2012	By PivotLearning: “@davidwees @lookforsun @mbteach What grade level do you think students would be able to self-regulate for online learning? #edchat”
2013	By edutopia: “Interesting read. MT @TechnologyToday: Self-regulation technique helps students focus in class: http://t.co/GMDBCShnoq #ntchat #edchat”
2014	By knowledgequest: “Self-regulation is not self-control: @StuartShanker #bced #edchat #sd61learn http://t.co/PKvYw8ZRvR”
2015	By utafrith: “You may have suspected it, but here is evidence: Self-regulated learning can be undermined by rewards. http://t.co/kPVdefeRZM”
2016	By misscs_teach: “Peri Peri Challenge.excellent recap of topic, encourages challenge and self-regulated learning #PedagooFriday https://t.co/dLy1rNweVQ”
2017	By Dylanwiliam: “Activities promoting self-regulated learning may be more effective in individualistic than collectivist cultures https://t.co/jUF2gpviZv”
2018	By MindShiftKQED: “This is when we want them to be challenged and pushed because this is when we can develop advanced thinking, as well as self-regulation,” said @ldsteinberg https://t.co/XFwQJNXY3v #edchat #teens #hschat #parents #teaching”
2019	By MindShiftKQED: “I love the strategy of “eating the frog,” or doing the most difficult thing on your to-do list first, so everything else will feel easier @edutopia #edchat #executivefunction https://t.co/uKn5cQGRO8”
2020	By Kemguro: “We are faculty members from the University of Santo Tomas who are currently working on an independent research focused on the relationship of students’ online learning readiness and self-regulated learning”
2021	By edutopia: “SRL is much more than just learning strategies to regulate emotions.” It’s also learning how to learn. https://t.co/L0yKtzuoVK”

### Geocoding

**FIGURE 4:**
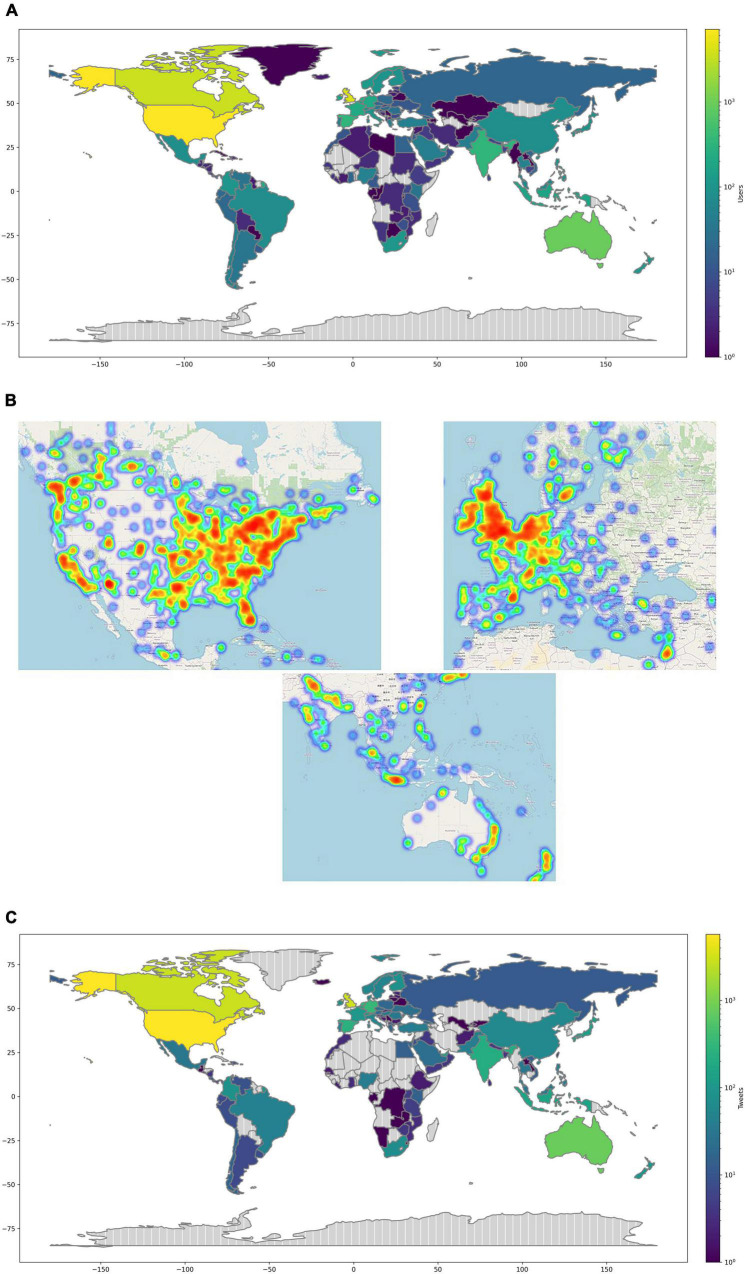
SRL geocoding stats. **(A)** Top, log scaled normalization of Twitter geotagged user. **(B)** Middle, from left to right, geotagged users heatmap of North America, Europe and the Middle East, and eastern Australasia. **(C)** Bottom, log scaled normalization of tweets per country.

In the geocoding analysis, we were able to classify 20,446 user origins out of the 29,556 users from the dataset, identifying 154 countries. Users in Twitter are meant to be who engaged within the dataset including those who tweeted, retweeted, liked, replied, quoted, or hashtagged. As mentioned earlier in the Geocoding section in the methodology, we used NLP techniques to identify Twitter users self-reported free text locations. Figure 4A depicts a normalized view of Twitter geotagged user distribution. The results show that the number of users differs among the countries. The top 10 countries with the highest number of geotagged users are United States (7,149 users), United Kingdom (4,268 users), Canada (3545 users), Australia (942 users), Spain (332 users), India (332 users), Netherlands (282 users), France (211 users), Germany (203 users), and Ireland (181 users). For those who stated their cities in their profiles, the geocoding analysis reports the following top cities London (704 users), Toronto (560 users), New York (312 users), Sydney (217 users), and Melbourne (200 users).

To get a more detailed view of the geographical distribution of Twitter users, we analyzed data from the major continents of the world (see Figure 4B). For North America, with the exception of New York, cities of Canada are leading Twitter microblogging on SRL. Cities of Toronto (560 users), Ottawa (199 users), and Vancouver (184 users) are among the highest. New York (312 users), Washington (180 users), and Los Angeles (123 users) lead US tweeting on SRL. In Europe, the geocoding analysis shows that London (704 users) scores the highest number of Twitter users of all cities and Europe. Other major cities are also from the United Kingdom, namely Birmingham (142 users) and Manchester (96 users).

Concerning Africa and Australasia, cities of Australia are placed on the top of the number of users, such as Sydney (217 users) and Melbourne (200 users). Some other cities from Asia are Dubai (60 users) and New Delhi (51 users). From Africa and South America are Bogotá (30 users) and Cape town (26 users).

We also looked at another variable of interest, namely the number of SRL-related tweets per country as seen in a normalized view in Figure 4C. The results of this figure align primarily with the outcome from Figure 4A except for a relevant appearance of two more Asian countries. The most tweeting countries are as follows, United States (5,712 users), United Kingdom (3,201 users), Canada (2,816 users), Australia (803 users), Germany (326 users), Netherlands (302 users), Spain (255 users), India (209 users), United Arab Emirates (154 users), and Indonesia (154 users).

For more information on other metrics per country, see Supplementary Material of this article.

### Topic Modeling

#### Coherence and the Number of Topics

**FIGURE 5:**
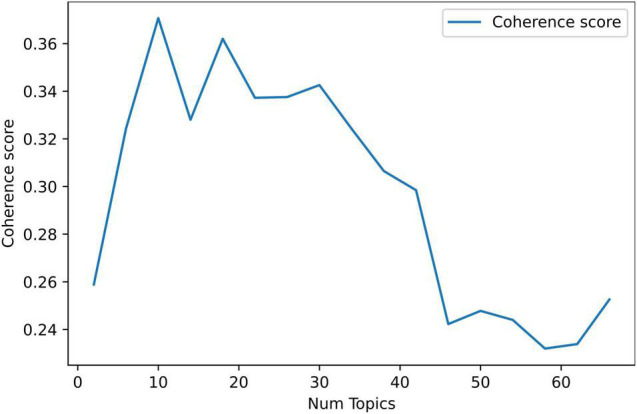
Coherence score for optimum number of topics in the topic modeling (retweets excluded).

At the first step of the topic modeling, we initially looked at identifying a number of topics. Such a process requires further testing of each number to detect the optimal value using the coherence score (Stevens et al., 2012). We used Gensim’s model for that purpose. The highest coherence score means the best word co-occurrence consistency. In order for us to differentiate the coherence value of each topic, we trained the topic modeling of LDA and examined up to 68 topics. At the end of the process of models training, we identified that 10 topics are the optimal number. As Figure 5 depicts, the coherence score (0.3707) was at the highest value on 10 topics, declining strongly after that, then became again higher at 18 topics (0.362). Nevertheless, 10 topics appear to be reasonable for our dataset size (Risch, 2016).

#### Themes and Topics

After addressing the number of topics to 10, we ran the LDA model and set the λ = 1 (lambda means that the relevance is defined entirely by appearing keywords). While we attempted to name the generated topics automatically, we discovered that the automatic labeling results are imprecise because of the scarcity of proper dictionaries on SRL and the need for manual edit knowledge. This result aligns with the studies by Lau et al. (2011) and Qiang et al. (2017), who stress that automatic labeling does not guarantee coherence. To produce meaningful results from the topic modeling, we decided to manually name themes that incorporate common topics based on the intertopic distance map, in spite of being more labor intensive. Themes were named based on the content of the top 30 words of each topic and the consensus judgment of the authors. To ease human intervention of identifying themes, the most common appearing words such as “child” and “skills,” which show up in several generated topics, were excluded.

**FIGURE 6:**
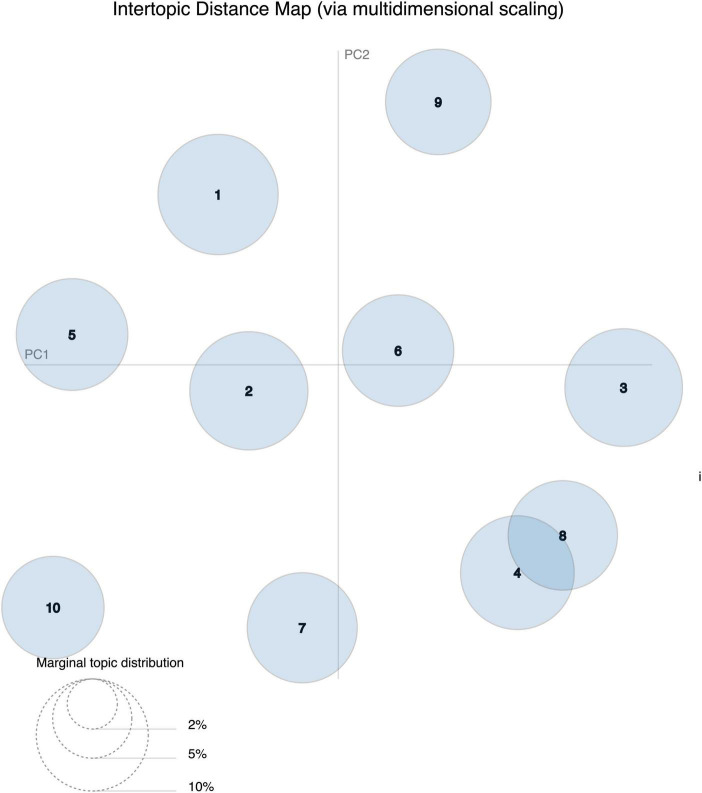
Intertopic distance map (*N* = 10 topics, retweets excluded). PC, principal component.

**FIGURE 7:**
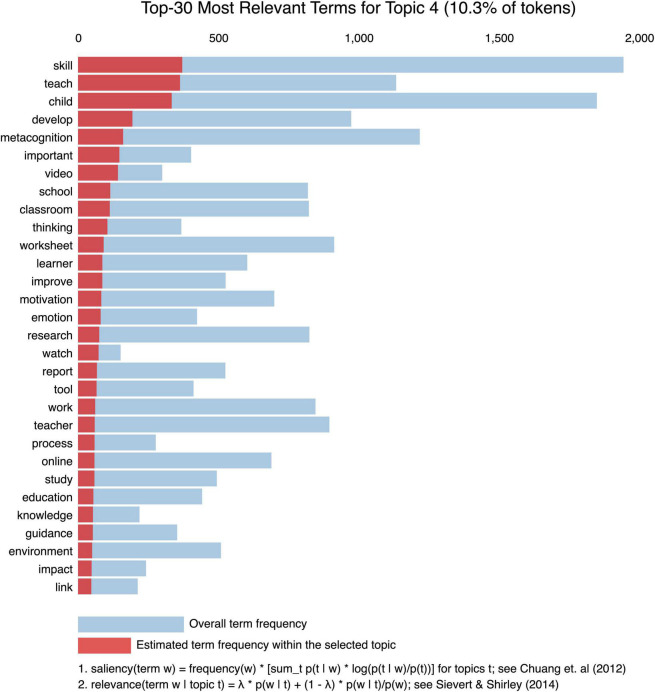
Most relevant terms for topic 4 (10.3% of the tokens).

Figures 6, 7 depict Gensim’s model through sets of visualizations. Figure 6 shows the map design of the topic model, in which 10 divergent topics are plotted as circles. The zone of the circles designates the general prevalence, and the center of the circles is determined by computing the distance between topics (Chuang et al., 2012). The intertopic distances are depicted on a 2D plane via multidimensional scaling. The principal component 1 (PC1) represents the transverse axis, and the PC2 represents the longitudinal axis. It is noteworthy that some of these topics overlap within the same dimensional scaling, like topic 4 and topic 8 which include keywords related to development and guidance. Other topics are entirely far away like topic 7 that has keywords on assessment and topic 10 which consists of words related to behavior and self-control. Yet, we matched the topics (i.e., through human intervention) into themes corresponding to similarity of the keywords. We followed the results of the intertopic distance map but also qualitatively agreed the themes together and gave relevant names grounding in the SRL tweets. Finally, the overall themes are labeled as the following: communication and help seeking (3 topics), self-control (2 topics), mindfulness (2 topics), online workshops (2 topics), and assessment (1 topic); The results of the topic modeling indicate some common appearing words in all the topics such as “child,” “skills,” “read,” “strategy,” and “metacognition.” See Table 5 for a detailed view.

**TABLE 5:** Topic classification, keywords, and size based (common appearing words excluded).

Theme	Topic	Keywords*	Size
Communication and help seeking	T3	Task, sequence, read, management, guidance, communication, check, knowledge, conversation, check	11%
	T4	Develop, video, important, study, online, guidance, improve, watch, report, tool	10.3%
	T8	Develop, thinking, social, report, read, guidance, time, early, circle, activity	9.5%
Self-control	T2	Promote, control, read, high, report, training, course, study, model, assessment	11.2%
	T10	Guide, calm, build, learner, work, start, behavior, improve, session, report	8.3%
Mindfulness	T5	Stress, goal, development, practice, work, study, foster, share, read, time	9.9%
	T6	Calm, develop, online, encourage, achievement, read, theory, tool, session, promote	9.9%
Online workshops	T1	Work, article, practice, share, join, develop, success, academic, blog, impact	11.4%
	T9	Research, group, tool, bring, foster, online, learner, share, digital, thinking, promote	8.9%
Assessment	T7	Worksheet, article, assessment, develop, tool, time, formative, design, environment, idea	9.6%

*Keywords are selected randomly from the pool of the top 30 words in each topic.

Figure 7 shows an example of the relevant top 30 keywords of topic 4. The coding results for all the topics are available in Supplementary Material.

## Discussion

In this work, we made a bold attempt to leverage open social network discussions from Twitter on SRL in the time period of 2011 till 2021. The analyzed dataset consists of 54,070 tweets created by 29,556 users and the current reporting on the analyses provides three main findings that answer the following research questions.

### What Are the General Characteristics of Twitter Conversation on Self-Regulated Learning?

The descriptive analysis showed a “moderate” increasing interest by the social media population of Twitter on SRL. That is, the breakdown of the dataset revealed a quite relevant tweet growth from 2011 until 2017 and a strong positive skew in 2018 (280% increase than 2017). Nevertheless, the analysis shows that the tweets markedly decrease as time goes by. The result of which the rate of fluctuation of the volume of the organic tweets, retweets, and engagements such as the number of hashtags and likes of SRL, do not quite align with the promising thoughts of academic researchers from the various fields of education who reported that the usage of SRL is garnered to be more popular (Paris and Paris, 2001; Matcha et al., 2019; Khalil, 2022). Perhaps, this conclusion is not a definite assumption given that discussions of SRL might tend to be more academic-oriented (e.g., conferences, research) than non-academic (professional, business, applied), however, not yet documented in previous studies to date. As a result, the public discussions on Twitter that cover SRL might be more allocated in other sources of information. Still, more retweets than organic tweets starting from 2017, suggesting a broader network of members sharing ideas from individuals. This decrease of organic tweets and the increase of retweets my denote some kind of a trend where users tend to shift toward resharing of content, showcasing more amplification of content and ideas (Greenhalgh et al., 2020).

The spike in the number of tweets from 2017 till 2018 could have a technical interpretation because of the extended character count limit from 140 to 280 characters in November 2017. As explained by Gligorić et al. (2018), users dealt with the character limit in different ways. Nevertheless, the drop of tweets in general after 2018 does not necessarily mean users should tweet more but could have led to more engagement in terms of more likes, retweets, and hashtags, as depicted in Figure 1.

Another interesting finding that we realize is the breadth of hashtags that our dataset addresses. It seems to be that there are education communities (e.g., #edchat) who contribute to discussions on SRL. This space is important for scholars to explore (see for e.g., the study by Staudt Willet, 2019) given that our examination of some of the linked tweets provides interesting points of view from practitioners and experts aligning well with Gee’s (2012) theoretical framework on affinity spaces. One more implication of this finding is that such spaces enable asynchronous communication unlike educational meetings that require a pre-set time. Interested parties on SRL may collaborate with new networks and engage in further conversations and exchange ideas (Manno, 2012). However, as Staudt Willet (2019) and Carpenter et al. (2020) discussed, it might be challenging for novices interested in a certain educational topic and looking into the Twittersphere to move their taste to hashtags like “#edchat.” Staudt Willet (2019) infers this challenge to the complex and overwhelming of information they could face as an entry point to new topics.

As academic scholars, we were interested in exploring hashtags relevant to academic venues. We found few hashtags linked to conferences, such as “#icalt2011,” the International Conference on Advanced Learning Technologies. There might be two explanations for the low representation of academic venues. First, the trend of which at academic venues, microblogging streams strongly at particular time spans and relatively stops when events finish. On the contrary, other discussions may sustain for longer periods, such as “#edchat,” and “#edtech.” The second explanation is that the tweets of SRL could implicitly appear in other contexts that are not directly used by tweeters in venues.

### What Are the Main Topics of Interest That Are Related to Self-Regulated Learning From Twitter Public Discussion?

Concerning the second research question, we relied on the word cloud of the most common words from the dataset as well as the computational process of topic modeling to provide an insight into the topics. Both analyses showed that there are common words sharing terms alike.

It was noticeable that words related to “child” are much more apparent in the context of the tweets. When investigated, it seems that discussions into employing self-regulation and interventions of self-monitoring are important for pupils at schools (Reid et al., 2005). Reid et al. (2005) explain that self-regulation is proposed as “an effective and efficient means for increasing students’ attention and academic productivity” (p. 361). In this work, we clearly understand that SRL is a key part of self-regulation. Apparently, the collected tweets included both and users were using them interchangeably.

Moreover, the word cloud has a strong demonstration of the words “metacognitive” and “metacognition” in the last few years. As commonly known, metacognition is central to SRL. Yet, it is interesting to see that metacognition is relatively mentioned than other terms, for example efficacy and awareness. Perhaps this aligns with the recent calls for focusing more on metacognition as a key trait for successful learners of SRL (Zhang and Zhang, 2019).

Both the topic modeling and word cloud sustain for repetitive demonstration of “skill” and “strategy.” According to Zimmerman (1990), SRL is a set of skills and strategies that guide learners’ future study and work paths. Twitter discussions are consistent with the further calls for improving these skills to succeed. Still, the frequent mentions of skills might imply time management, awareness, and self-monitoring which were not relatively considered by the Twitter users in the dataset.

Building on the strong coherence value, the topic modeling of the tweets resulted in 10 topics which were then organized into 5 themes. It is worth to note that this quantitative approach of algorithmic categorization has its own limitation, therefore inappropriate to generalize the results (see section “Limitations and Future Direction” for more details). The bigger theme which accounts for nearly 31% of the clusters size indicates a growing discussion on communication and asking for help. Relatively appearing words of “communication, help, guidance, worksheet” suggests that help-seeking, which is commonly known as an important skill for SRL (Karabenick and Berger, 2013), is well connected with communication. The open sphere of Twitter may have implied the growth of topics in relation.

An interesting theme that we allocated is mindfulness. This theme came at a surprise when other key terms did not exist in sight (e.g., self-evaluation). Our analysis of this finding is consistent with prior research which found out that Twitter functions for venting out one’s stress (Doan et al., 2017). In relation to education, stress might be a result of exam burdens and mismanagement. Withal, SRL and mindfulness has been proven to help students to cope up with stress (Ramli et al., 2018). It is possible that bloggers, including students, have used Twitter space to reflect upon stress and bad management. As some studies like (Carpenter et al., 2020) discovered that users valued Twitter to reduce not only stress, but also isolation, the appearance of this theme suggests a very interesting area for scholars to further investigate.

The prevalent words formation of “time,” “management,” and “metacognition” in the topic modeling may provide valuable insight on planning (Wong et al., 2019) and academic procrastination (Limone et al., 2020). This is an interesting area of research in SRL since several studies have found that SRL can provide the needed skills to reach wise decisions and solve problems which is positively correlated not only to one’s academic life, but also to wellbeing (Limone et al., 2020). The same derived words may suggest associated talk on anxiety despite the word itself has not emerged in our context but linked to discussions on stress as previously mentioned. Apart from that, we also labeled Online workshops for two topics from the topic modeling. We looked at some of the tweets in this theme and found out that several share disseminating of information in conferences and workshops. Others communicated workshops to foster metacognitive skills in technology-domain settings. This finding shed considerable light on how the Twitter community facilitate supporting SRL and contribute to disseminating SRL research, practices and work.

Even though self-evaluation is imperative for SRL in social media channels (Yen et al., 2021), relevant words and topics to self-evaluation were nearly absent from the topic modeling and word cloud analysis of the tweets. This finding coordinates well with the systematic review study by van Houten-Schat et al. (2018), who found out that there is a dearth of SRL interventions on self-evaluation for students. This may suggest a gap that triggers interest for further exploration by those interested in SRL theory.

Finally, further inspection of the topics uncovers two areas from the public discussions on Twitter, self-control and assessment. Both themes might be quite linked to each other, as seen in Figure 6 and as has been reported by Zhu et al. (2016). The link of which self-control may have a “significant impact” on students’ performance (ibid). As an implication, we foresee those discussions around self-control and SRL together with assessment could be predicted to be an intriguing forum on Twitter.

### Where Do the English-Based Self-Regulated Learning Discussions Originate From?

With the expansion of the Internet, social media and the Web, social media analytics is a key tool that function not only as a support to diverse decision systems, but also as a geographic location data revealing how microblogging data can be used at different scales.

To answer our third research questions, the geocoding analysis unveiled interesting results. The presence of high-density geotagged users in some particular countries was obvious from the heatmap shown in Figure 4. One of the main findings is that most of the tweets on SRL originate from the Global North rather than the Global South countries, with some exceptions. For example, some Global South countries such as India, Indonesia, Mexico, and Brazil show a high density of users who tweet on SRL. Provided that these countries are the largest in the Global South in terms of population, it is fairly more common to reach this outcome. However, some countries in the Global South were not as active as the ones mentioned, such as China. Our interpretation of this low presence aligns with other studies such as Wu et al. (2016) which report that many countries use an assortment of censors and blockage means to disrupt sharing of sensitive information over the social network services. Users from some countries like North Korea and China might use Virtual Private Networks (VPNs) to overcome firewalls. Therefore, the geocoding analysis identified this low representation. For Africa and South American continents, relatively fair traffic produced from the countries of South Africa and Colombia suggests that SRL awareness is expanding and becoming more diverse.

For the Global North, we discovered that some geographic regions are not as active as the others. Speaking of which, the user representation of the United States, United Kingdom, and Canada alone accounted for over 50% of the total number of users. The other half goes for the rest of the countries. This presence of a high density of tweets of only three countries of the world was unexpected. Perhaps an explanation for such a finding relates to the popularity of Twitter in these countries (Wu et al., 2016). However, it could also be explained that SRL research and discussions are more popular in these geographical regions given that known theories and practices on SRL such as Zimmerman (1990) and Winne and Perry (2000) originate from these particular areas. Another common point between the United States, United Kingdom, and Canada countries is that 50% of the geotagged users derive from English native speaking countries, which could be another interpretation for this particular outcome.

The geocoding results from this study may raise concerns on the dominance of self-regulation and SRL discussions by high-income countries, thus limiting context on the theory from many other parts of the world. This issue has been previously discussed by Haslam et al. (2019), who found out that high-income countries have conducted the majority of research studies and discussions on self-regulation. Although SRL is one domain of self-regulation, our results from geocoding match Haslam et al.’s (2019) conclusion. We argue that more insights are needed from low and middle-income countries to draw a broader perspective on SRL.

## Conclusion

This research provides useful insights by analyzing Twitter discussions on SRL from various perspectives of geolocation, content, hashtags, users, topics, and themes. Considering 54,070 tweets and 29,556 users, we introduced and provided a broader understanding of public discussions on SRL from a relatively new research direction (i.e., Twitter) in the last 10 years.

The approach of conducting this research allowed us to explore a wider public view on SRL and highlighted some interesting insights on content, topics, and geolocations. We conclude that there are intriguing public discussions on SRL and communities interested in knowing more and discussing SRL strategies outside scholarly venues. However, the recent drop in tweets in the last couple of years (may) conclude a lack of interest in SRL for several reasons discussed in the study.

We conclude that topic modeling with LDA inferred different topics and aspects of communication, help-seeking, mindfulness, workshops, self-control and assessment, yet with differed tweet quantities per each. Also and even though estimating the location of the users from our dataset was a non-trivial task, geocoding of the SRL tweets has provided us with new insights that socioeconomic gradients, technology advancement, and theory-originating of some countries may have affected the higher density of geotagged users from high-income countries and the Global North than the Global South. This dominance of discussion on Twitter concludes that SRL and its wide domain of perceived subtopics (e.g., self-control, self-evaluation, goal settings) might be reticent by particular countries, thus confounded.

The research work also contributes to established research on using Twitter as an affinity space and extends this exploration by quantifying contents using computational processes of topic modeling, geocoding and descriptive analyses.

Last but not least, this research opens up several questions to be further explored by practitioners and scholars, such as self-evaluation and the mindfulness domain (e.g., stress, anxiety).

### Implications

Further research can use the identified topics and analysis to understand SRL from a different perspective. Indeed, the ideology of using affinity spaces on Twitter may provide a hospitable sphere to find interesting thoughts that are outside typical scholarly publications (i.e., book and research papers) and static medium. Scholars, teachers and practitioners can tap affinity spaces on Twitter to chase diverse sources of information on SRL. Additionally, through the analysis, we found that social media may allow novices to take advantage of microblogs and the vast experiences available from a larger pool of fellow scholars and educators and enrich their own (Carpenter et al., 2020). A likewise practical implication we distill from this study is that the generated topics based on the topic modeling and the most commonly appearing words show that Tweets suggest new areas that demand further SRL examination. This implication aligns with (Marcelo and Marcelo, 2021), who found that particular types of users, such as influencers, may act as “knowledge brokers” and intermediate content by sharing and creating elegant materials on SRL.

Finally, our work has also potential implications related to the geographical distribution of interest on SRL on Twitter. The geographical disparities in Twitter’s microblogging from specific parts of the worlds require further bridging between the Global North and the Global South on knowledge and expertise exchange on SRL. Provided the broad growth of this theory, there is a persistent need to invite scholars, educators, and practitioners to a global virtual carnival of knowledge share not only from a specific region, but from all over the world.

## Limitations and Future Direction

This work incurs some limitations. Because the research study has been carried out on a social network service, the current limitations may have affected our overview of the research results. First of all, even though the dataset is not very large as we expected, it is worth noting that perhaps some tweets were not mined by the Twitter API. This might be due to that fact that some tweets were deleted right after being posted by the user. Another related aspect, the Twitter API may have also mined some tweets that were already been deleted by users at a later stage, therefore, this study may have considered tweets despite being deleted by their owner(s).

Second, the used search terms may have failed to crawl SRL tweets as intended to be. That is, some users may have used a venue or specific hashtags that are not used in the search keywords. In a related aspect to the limitations to the search terms, the trigram search of “self-regulated learning” may have scrapped some tweets that are irrelevant to the context of the study. Also, our main search terms were done in English, as a consequence, we may have missed some important microblogs that are relevant to this study.

The third and fourth limitations of this study are related to the geocoding and topic modeling analyses. For geocoding, even though we tried to show the locations of the users who tweet specifically on SRL, the absence of some countries such as China and Russia was notable. Such causes are a common practice given the complications between US politics and some other countries; for further elaboration on the issues see Twitter Safety (2020). Another limitation of our geocoding analysis is the sampling bias since the collected tweets are originated from English-based search term. Finally, the topic modeling may have exaggerated specific phenomena while overlooking other ones. However, we tried to overcome this issue by qualitatively examining the top 30 words of each topic and introducing white and black lists. Still, human intervention was required to choose the best model, name themes from topics, and depict results in a meaningful manner.

As a future direction of this work, we may improve the data collection process by examining academic papers, mainly on SRL, building an additional list of complementary keywords from the SRL theory, and then searching specifically for wordings and hashtags consisting of such. In this case, we can increase the size of our dataset, and by then, our conclusions can be more accurate. Another future direction is related the geocoding analysis. We plan to normalize the total number of tweets and link that to the country population. As a result, we foresee that representation of the countries on the heatmap (as shown in Figure 4) will be more accurate and less biased. Furthermore, the analysis can be extended to other social media platforms for better mapping and understanding of self-regulation.

## Data Availability Statement

The analysis script source code of the topic modeling and the word cloud is available on GitHub (https://gist.github.com/slate-dev/8f59772a790e5a3ed70788fab70d5343). The datasets analyzed for the geocoding in this study can be found in Datasheet 1.CSV and Datasheet 2.CSV. All research metrics per country are available in Datasheet 3.CSV. Tweet ids are available in Datasheet 4.CSV. The overall topic modeling analysis code results are available in Datasheet 5.CSV.

## Ethics Statement

Ethical review and approval was not required for the study on human participants in accordance with the local legislation and institutional requirements. Written informed consent for participation was not required for this study in accordance with the national legislation and the institutional requirements.

## Author Contributions

MK conceived the presented idea, carried out analysis, verified the analytical methods, interpretation of the results, and handled writing the whole article. GB carried out the data collection, analysis, and further discussions. Both authors contributed to the article and approved the submitted version.

## Conflict of Interest

The authors declare that the research was conducted in the absence of any commercial or financial relationships that could be construed as a potential conflict of interest.

## Publisher’s Note

All claims expressed in this article are solely those of the authors and do not necessarily represent those of their affiliated organizations, or those of the publisher, the editors and the reviewers. Any product that may be evaluated in this article, or claim that may be made by its manufacturer, is not guaranteed or endorsed by the publisher.

## Footnotes

1 For more information, see https://docs.python.org/3/library/ (last accessed October 2021).

2 For more information on search patterns of regex, see https://en.wikipedia.org/wiki/Regular_expression (last accessed October 2021).

3 https://twitter.com/TwitterSupport/status/1141039841993355264?s=20 (last accessed January 2022).

4 Open-source library for NLP.

5 Open-source geocoding service.

6 https://github.com/mediagis/nominatim-docker (last accessed August 2021).

7 https://teach.com/blog/what-is-edchat/ (last accessed November 2021).
